# Microbial gut evaluation in an angolan paediatric population with sickle cell disease

**DOI:** 10.1111/jcmm.17402

**Published:** 2022-09-28

**Authors:** Mariana Delgadinho, Catarina Ginete, Brígida Santos, Joana Mendes, Armandina Miranda, Jocelyne Vasconcelos, Miguel Brito

**Affiliations:** ^1^ H&TRC‐ Health & Technology Research Center, ESTeSL‐ Escola Superior de Tecnologia da Saúde Instituto Politécnico de Lisboa Lisbon Portugal; ^2^ Centro de Investigação em Saúde de Angola (CISA) Bengo Angola; ^3^ Hospital Pediátrico David Bernardino (HPDB) Luanda Angola; ^4^ Instituto Nacional de Saúde Doutor Ricardo Jorge (INSA) Lisbon Portugal

**Keywords:** 16S rRNA, foetal haemoglobin, microbiome, sickle cell disease

## Abstract

Sickle cell disease (SCD) is one of the most common genetic conditions worldwide. It can contribute up to 90% of under‐5 mortality in sub‐Saharan Africa. Clinical manifestations are very heterogeneous, and the intestinal microbiome appears to be crucial in the modulation of inflammation, cell adhesion and induction of aged neutrophils, the main interveners of recurrent vaso‐occlusive crisis. Enterocyte injury, increased permeability, altered microbial composition and bacterial overgrowth have all been documented as microbial and pathophysiologic changes in the gut microbiome of SCD patients in recent studies. Our aim was to sequence the bacterial 16S rRNA gene in order to characterize the gut microbiome of Angolan children with SCA and healthy siblings as a control. A total of 72 stool samples were obtained from children between 3 and 14 years old. Our data showed that the two groups exhibit some notable differences in microbiota relative abundance at different classification levels. Children with SCA have a higher number of the phylum Actinobacteria. As for the genus level, *Clostridium* cluster XI bacteria was more prevalent in the SCA children, whereas the siblings had a higher abundance of *Blautia, Aestuariispira, Campylobacter, Helicobacter, Polaribacter* and *Anaerorhabdus*. In this study, we have presented the first microbiota analysis in an Angolan paediatric population with SCD and provided a detailed view of the microbial differences between patients and healthy controls. There is still much to learn before fully relying on the therapeutic approaches for gut modulation, which is why more research in this field is crucial to making this a reality.

## 1 INTRODUCTION

Sickle cell disease (SCD) is a relatively common genetic disease, being a growing health problem in many parts of sub‐Saharan Africa, and about 300,000 affected children are born with this condition every year.^1^ In Africa, the mortality rate among SCD children under the age of 5 years can be as high as 90%.^2^ This Mendelian disease results from a single‐nucleotide polymorphism (rs334), which changes the position 17 of HBB gene from thymine to adenine, resulting in the replacement of glutamic acid to valine residue (Glu6Val) at position 6 of the haemoglobin chain.^3^, ^4^ It is usually recognized as an autosomal recessive disorder, where carriers (HbAS) are normally asymptomatic, whereas homozygotes (HbSS) suffer from sickle cell anaemia (SCA).^5^, ^6^, ^7^ SCA is a lifelong disease characterized by chronic haemolytic anaemia, unpredictably painful episodes and widespread organ damage that leads to premature death.^5^ The sickle erythrocytes can often cause vaso‐occlusive crises (VOC), which are one of the most variable and frequent complications in this disease, whether in terms of intensity, chronicity or frequency.^5^, ^8^ Among the SCA patients, there is substantial phenotypic heterogeneity which supports the idea that other factors play an important role in the disease modulation.^9^ Given the clinical heterogeneity experienced by the patients, finding new treatments more efficient and with fewer side effects is essential. One promising therapy could be gut microbial modulation in order to mitigate symptoms.

Recent studies have reported several pathophysiologic and microbial changes in the intestines of SCD patients, and these can include the following: increased permeability, enterocyte injury, altered microbial composition and bacterial overgrowth.^10^ The gut microbiome seems to be crucial in the modulation of inflammation, cell adhesion and production of aged neutrophils, which are the main interveners of recurrent VOC episodes.^11^ Since intestinal microbes can regulate aged neutrophils, abnormalities in either the integrity of the intestinal barriers or chronic disequilibrium of the intestinal microbiota are very likely to occur in SCD patients.^12^

A study performed in mice demonstrated that depletion of faecal microbiome reduces the number of circulating aged neutrophils with improvement of the pathogenesis and inflammation‐related organ damage in SCD models.^13^ Another recent study with SCD mice revealed that gut microbiome depletion with antibiotics leads to a reduction in inflammatory cytokines (TNF‐α, IL‐17, IL‐27 and IL‐10) and rescues bone loss.^14^

Moreover, there is evidence of reduced diversity and increased abundance of *Veillonella* in SCD intestinal microbiome, which correlates with VOC frequency.^15^ It has also been reported that SCD patients have reduced *Alistipes* and *Pseudobutyrivibrio*, both of which correlated negatively with serum lactate dehydrogenase, and it was found that SCD individuals with a higher abundance of *Lachnoclostridium* had higher haemoglobin values, elevated foetal haemoglobin levels and lower white cell count.^12^ Dutta and colleagues^16^ hypothesize the existence of a vicious cycle of VOC in sickle cell disease, defending that intestinal dysbiosis and injury observed in SCD subjects cause a breach in the gut barrier and VOC also directly induces intestinal injury and increases gut permeability. Then, this compromised gut barrier will facilitate the translocation of intestinal bacteria and bacterial products into the bloodstream, where the microbes or their products would not be enough to cause overt infections, but sufficient to increase neutrophil activation and ageing, which will feed into the vicious cycle once again and facilitate further VOC occurrences.^12^, ^16^

All the studies mentioned above support the idea that intestinal microbial composition in SCD populations is unquestionably altered, but there is still much to learn before fully relying on the clinical approaches for gut modulation. Studying different SCD populations at various ages could provide us the answer we seek. With this in mind, our aim was to sequence the bacterial 16S rRNA gene in order to characterize the gut microbiome of Angolan SCA children and healthy siblings.

## 2 MATERIALS AND METHODS

### 2.1 Study Population

This cross‐sectional study included 72 Angolan children (36 SCA patients and 36 healthy siblings) aged between 3 and 14 years old. In the sibling group, 25 children were carriers (AS genotype) and 11 had the AA genotype. This project is part of a much wider study involving an Angolan SCD cohort. Half of our population were regularly attending consultations at ‘Hospital do Caxito’ in Bengo Province and the other half at ‘Hospital Pediátrico David Bernardino’ in Luanda. Inclusion criteria included no antibiotic exposure in the 3 months prior to sample collection.

Written informed consents were obtained from the children's legal guardians prior to inclusion in the study. The study was approved by the Ministry of Health of Angola (CE. N° 26/2020) and the Ethical Committee of ESTeSL (CE‐ESTeSL‐N°0.67/2021), and all methods were conducted in accordance with the guidelines of the Declaration of Helsinki. Moreover, an eating behaviour questionnaire was distributed to check whether there were major differences in food habits.

### 2.2 Sample collection and DNA extraction

A whole blood sample was collected from each SCA patient for haematological and biochemical analysis, electrophoresis for SCA confirmation and foetal haemoglobin quantification. From the siblings, a blood drop was collected on a filter paper, for the determination of the rs334 polymorphism.

Haematological determinations included complete blood count (erythrocytes, reticulocytes, white blood cells and platelets), haemoglobin, mean corpuscular volume (MCV) and mean corpuscular haemoglobin (MCH), which were determined using the XT‐2000i Haematology Analyser (Sysmex Corporation, Kobe, Japan). The haemoglobin fractions, including HbF, were quantified by ion‐exchange HPLC (Biorad Variant II).

Biochemical blood tests were determined using Cobas C111 (Roche Diagnostics, Basel, Switzerland) and Mindray BA‐88A (Mindray, Shenzhen, China) and included Lactate dehydrogenase (LDH), creatinine, urea, total and direct bilirubin, aspartate aminotransferase (AST) and alanine aminotransferase (ALT) levels.

Stool samples were collected using the DNA/RNA Shield Faecal Collection tubes (Zymo Research), which contain a preservation solution that ensures sample stability during transportation or storage. Metagenomic DNA was extracted using ZymoBIOMICS™ DNA Miniprep Kit (Zymo Research) according to the manufacturer's instructions, and FastPrep‐24™ homogenizer (MP Biomedicals) was used for cell lysis. DNA samples were quantified with NanoDrop™ One spectrophotometer (ThermoScientific) and stored at −20°C until processing.

### 2.3 16S Sequencing

After PCR amplification, the dsDNA HS assay kit for the Qubit 3.0 fluorometer (Thermo) and the TapeStation 4200 with the High Sensitivity D1000 ScreenTape and Reagents (Agilent) were used to measure DNA concentration and amplicon lengths, respectively. Tagmentation, clean‐up, normalization and pool of libraries were performed following the Nextera XT DNA Sample Preparation Reference Guide (Illumina) to generate paired‐end DNA libraries. Purification steps were carried out by using AMPure XP beads (Beckman Coulter), and all the resulting indexed libraries were checked on TapeStation. A sequencing run of 2 × 151 bp paired‐end reads was performed on the NextSeq 550 instrument (Illumina). After the procedure, the software generated analysis output in the FASTQ file format.

### 2.4 Bioinformatics and Statistical Analysis

The 16S Metagenomics app v1.1.0 within BaseSpace (Illumina) was used to perform taxonomic classification, which uses the RefSeq RDP 16S v3 database^18^ and the RDP Naïve Bayes taxonomic classification algorithm.^19^ Shannon indices for alpha diversity were calculated from OTU data and checked for statistical differences using Mann–Whitney U‐test.

Clinical data were analysed with SPSS version 27 (IBM), and significant differences between the patient's microbiota at various taxonomic levels were assessed using the Statistical Analysis of Metagenomic Profiles (STAMP) software package v2.1.3.^20^ The statistical significance was tested using Welch's test. *p*‐values <0.05 were considered as statistically significant.

## 3 RESULTS

### 3.1 Clinical characterization of the study population

As mentioned, 36 SCA patients and 36 healthy siblings participated in this study. The patient group comprised both genders (58% females), and patients ranged in age from 4 to 12 years old (mean of 7.97 ± 2.31 years). In the sibling group, 52% are female, and ages ranged from 3 to 14 years old (mean of 8.11 ± 3.61 years). The average age for the SCA diagnostic was 31.9 months, although the average for the first symptoms was 11.7 months. The most common first symptom was dactylitis (69.7%), followed by painful crisis (13.9%) and severe anaemia (16.7%). From the 36 patients, only 13.9% never had a transfusion (mean of transfusions 2.97 ± 2.94) and 11.1% have never been hospitalized (mean of hospitalizations 3.25 ± 2.77). Haemoglobin levels ranged from 5.07 to 9.33 g/dl (mean 7.23 ± 0.91 g/dl) and foetal haemoglobin levels from 0.5 to 16.1 (mean 4.1 ± 3.6%) (Table 1).

**TABLE 1:** Clinical characteristics of the patients with sickle cell anaemia (*n* = 36)

Variables	Mean	SD
Haemoglobin (g/dl)	7.2	±0.9
Foetal Haemoglobin (%)	4.1	±3.6
Erythrocytes (10^12^L)	2.9	±0.5
MCV (fL)	78.1	±7
MCH (pg)	25.6	±2.4
White blood cells count (10^9^L)	13.7	±3.6
Neutrophils count (10^9^L)	5.7	±1.9
Platelet count (10^9^L)	420.7	±173.7
Reticulocyte count (%)	10.6	±4.6
LDH (U/L)	516.2	±163.6
Creatinine (mg/dl)	0.5	±0.7
Bilirubin (mg/dl)	1.8	±1.6
Direct Bilirubin (mg/dl)	0.8	±0.7
Urea (mg/dl)	19.7	±14.7
GOT (U/L)	37.3	±26.2
GTP (U/L)	13.9	±14.3

**FIGURE 1:**
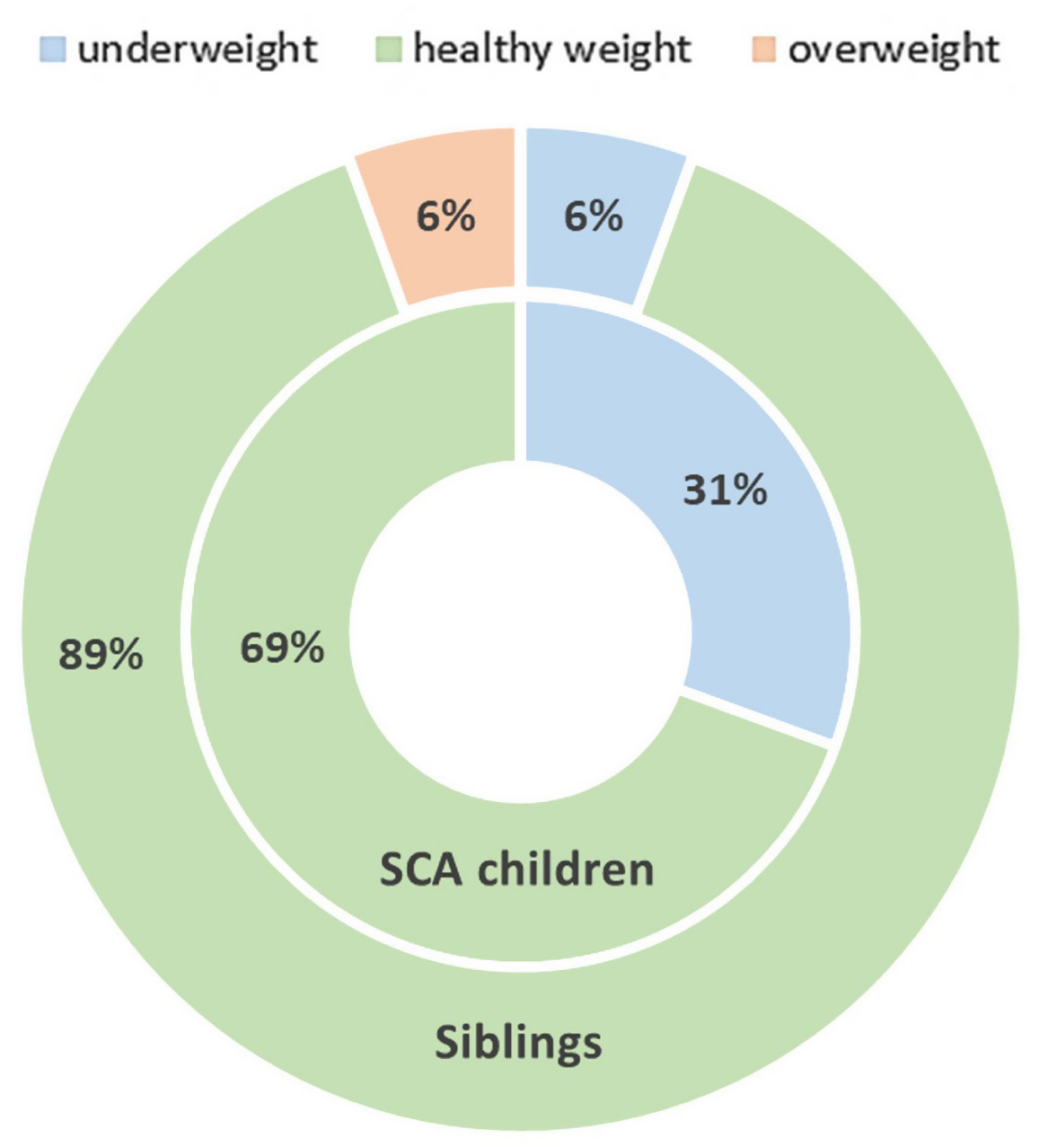
Prevalence of each BMI group classification in the studied population (*n* = 72)

The body mass index (BMI) percentile and BMI group were determined for both populations (Figure 1). The majority of the studied population had normal weight, although 31% of the SCA children and 6% of the healthy siblings are underweight, and 6% of the siblings are overweight. We obtain a *p*‐value of 0.003 in the BMI group and 0.049 in the BMI percentile using the Mann–Whitney test.

Sickle cell anaemia subjects were also retrospectively divided into two groups based on HbF values: less or higher than 5%. As expected, these two groups had significant differences in some clinical parameters, such as: the number of transfusions per year (*p* = 0.007), the frequency of hospitalizations per year (*p* = 0.017), G gamma: A gamma ratio (*p* = 0.013) and the number of malaria episodes (*p* = 0.003). All the studied SCA patients were naïve for Hydroxyurea.

### 3.2 Sequencing data

**FIGURE 2:**
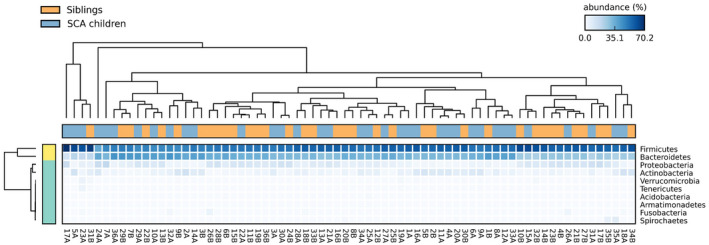
Heatmap of the 10 most prevalent bacterial phylum in the population. The colour intensity in each panel shows the relative abundance in percentage of each phylotype. Metagenomic samples were grouped by similarity and dendrograms were constructed by the average neighbour algorithm and unweighted pair group method with arithmetic mean (UPGMA)

A total of 76 stool samples were collected and analysed in this study. After sequencing, the samples yielded 42,775,839 quality‐filtered reads with an average of 594,109 reads per sample. In total, 5337 operational taxonomic units (OTUs) were identified and classified into 50 phyla, 123 classes and 235 orders. A mean of 82.8% reads were classified for the genus level, and an average of 1076 species were identified in our samples. Proteobacteria, Firmicutes, Bacteroidetes and Actinobacteria were the most abundant phyla of the total reads (Figure 2).

### 3.3 Phenotype and microbial diversity

**FIGURE 3:**
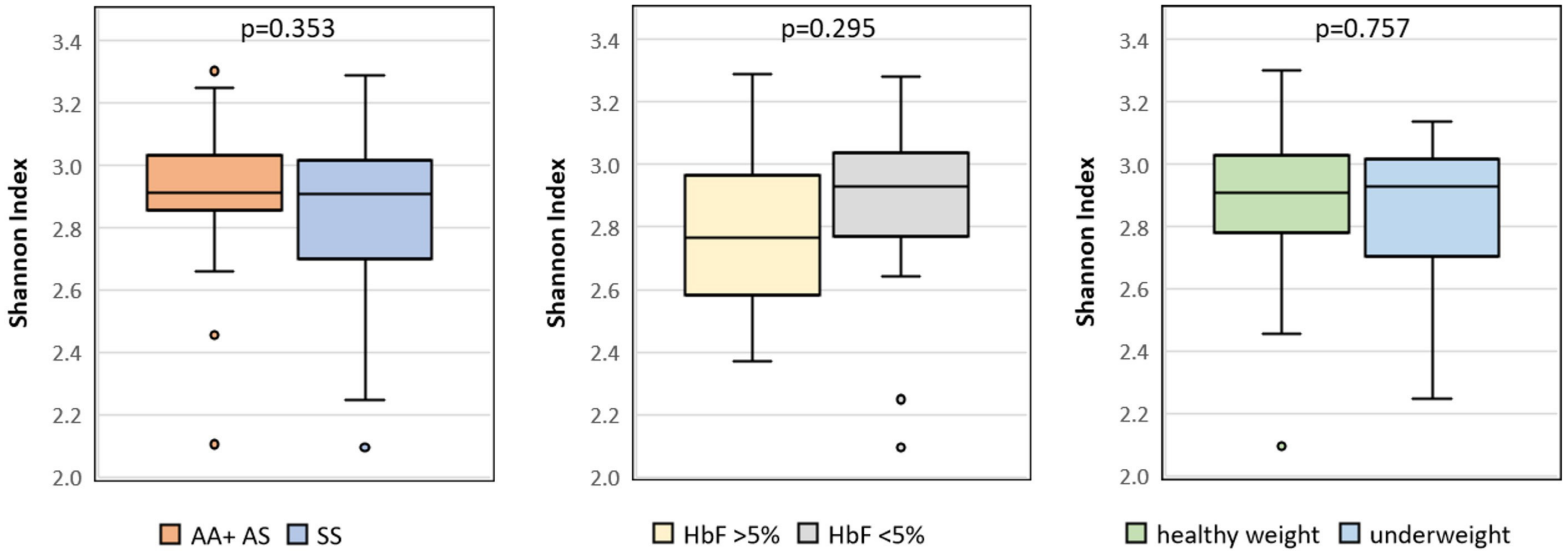
Boxplots of alpha diversity calculated by Shannon index, relatively to the: (A) Patient genotype group, siblings (AA+AS) or SCA children; (B) Foetal haemoglobin level and (C) BMI group. *p*‐values were calculated with Mann–Whitney *U*‐test

To explore the alpha diversity within the samples, we used Shannon index for comparing the genotype, HbF and BMI groups (Figure 3). Although we found some differences in these variables, none were statistically significant.

**FIGURE 4:**
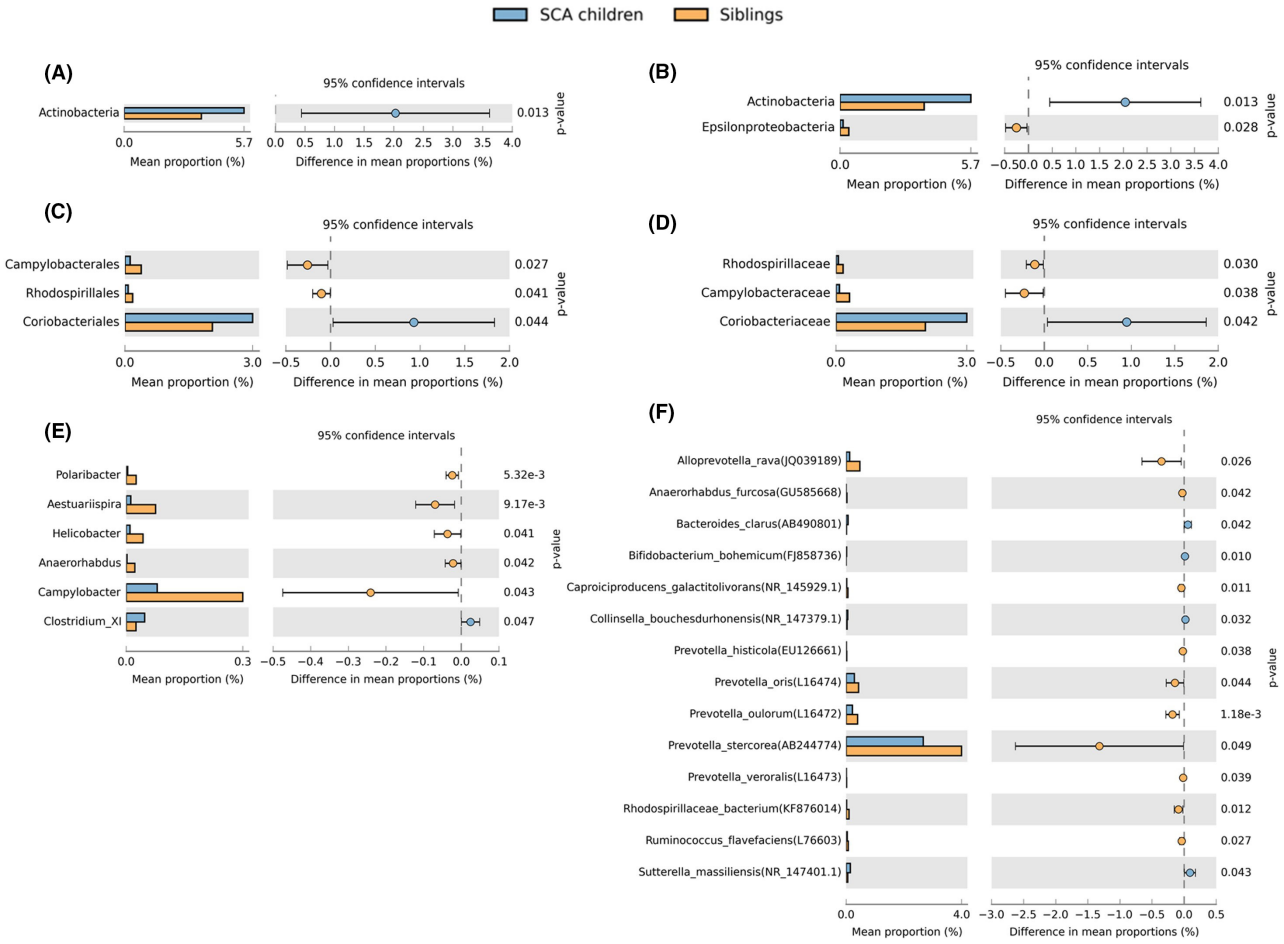
Distribution of metagenomic data in SCA children and healthy siblings for the levels of: (A) phylum (B) class (C) order (D) family (E) genus (F) species. The results were filtered using a p‐value of 0.05 with a Welch's *t*‐test, effect size of 0.01 threshold and removing unclassified reads in STAMP software. The difference in mean proportions indicates the mean proportion of certain bacteria in SCA children minus the mean proportion in siblings

The SCA and control samples show some notable differences in microbiota relative abundance, as a per cent of reads assigned, at different levels of classification (Figure 4). In particular, children with the disease have a higher number of Actinobacteria (*p* = 0.013), which is the fourth most prevalent phylum, with a mean of 5.47% (±3.49) whereas the siblings have a mean of 3.25% (±2.98) (Figure 4A). The Epsilonproteobacteria class showed a significant reduction in SCA (Figure 4B), the Campylobacterales and Rhodospirillales orders also showed a significant reduction in SCA, and Coriobacteriales showed a significant increase (Figure 4C). The *Coriobacteriaceae*, a bacterial family which belongs to the Actinobacteria phylum, also show significant differences (*p* = 0.042) with 2.05% (±1.80) in the controls and 3.00% (±2.03) in the SCA (Figure 4D). The *Rhodospirillaceae* and *Campylobacteraceae* are two other significant families, which belong both to the Proteobacteria phylum and appear to be more predominant in the sibling's group, *p* = 0.030 and *p* = 0.038, respectively (Figure 4D). As for the genus level (Figure 4E), only *Clostridium* cluster XI bacteria was more prevalent in the SCA children, whereas the siblings had higher numbers of *Aestuariispira, Campylobacter and Helicobacter,* all from Proteobacteria phylum, and *Polaribacter and Anaerorhabdus* both from Bacteroidetes phylum. In Figure 4F, it is possible to observe that the controls had higher numbers in five species of the *Prevotella* genus and the SCA had four species that were more prevalent: *Bacteroides clarus, Bifidobacterium bohemicum, Collinsella bouchesdurhonensis* and *Sutterella massiliensis*. Besides the species of *Prevotella*, the siblings had also higher numbers of *Alloprevotella rava, Anaerorhabdus furcosa, Caproiciproducens galactitolivorans, Rhodospirillaceae bacterium* and *Ruminococcus flavefaciens*.

**FIGURE 5:**
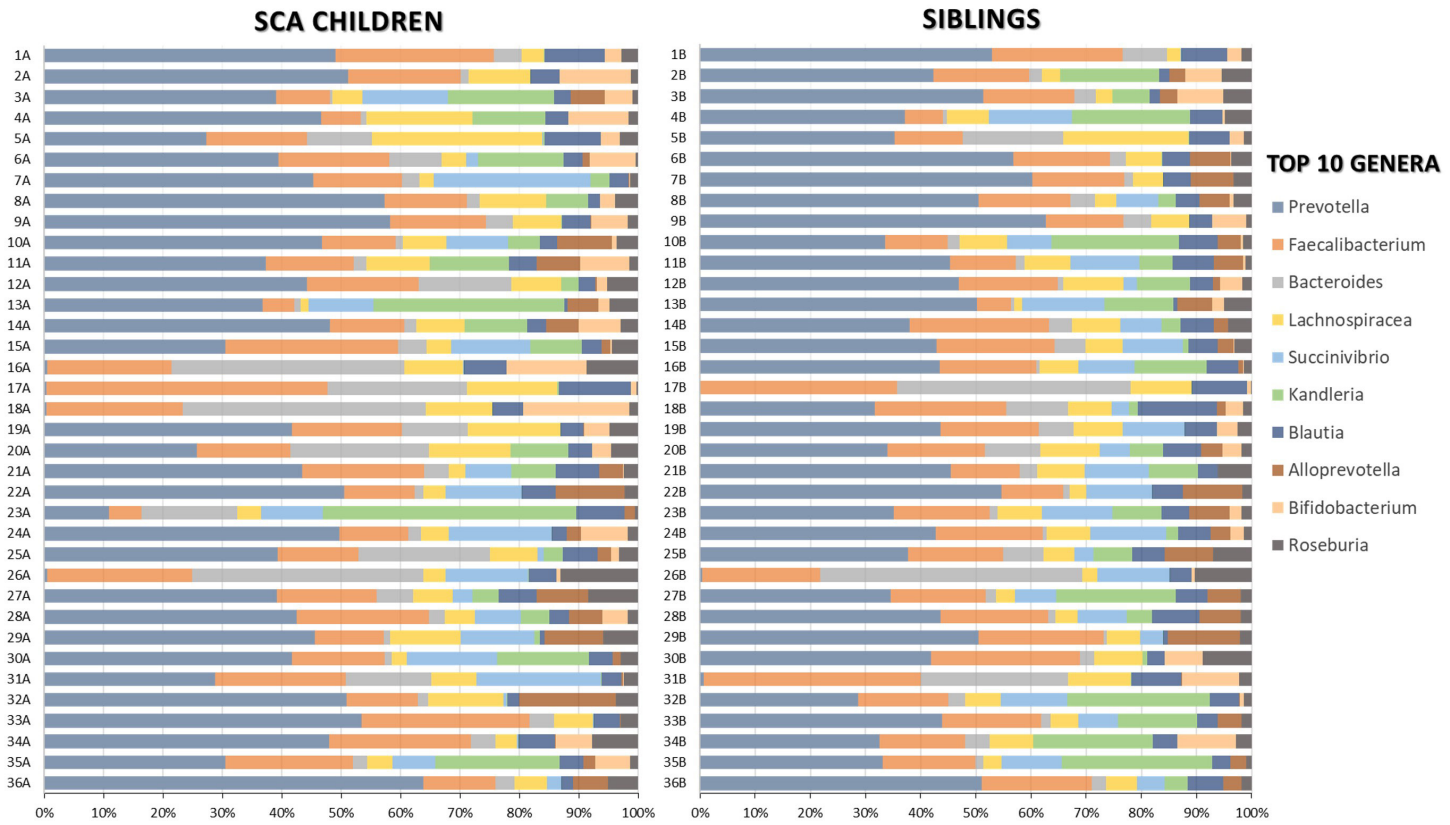
Relative abundance of the ten most prevalent bacterial genera between SCA children and corresponding healthy siblings

**FIGURE 6:**
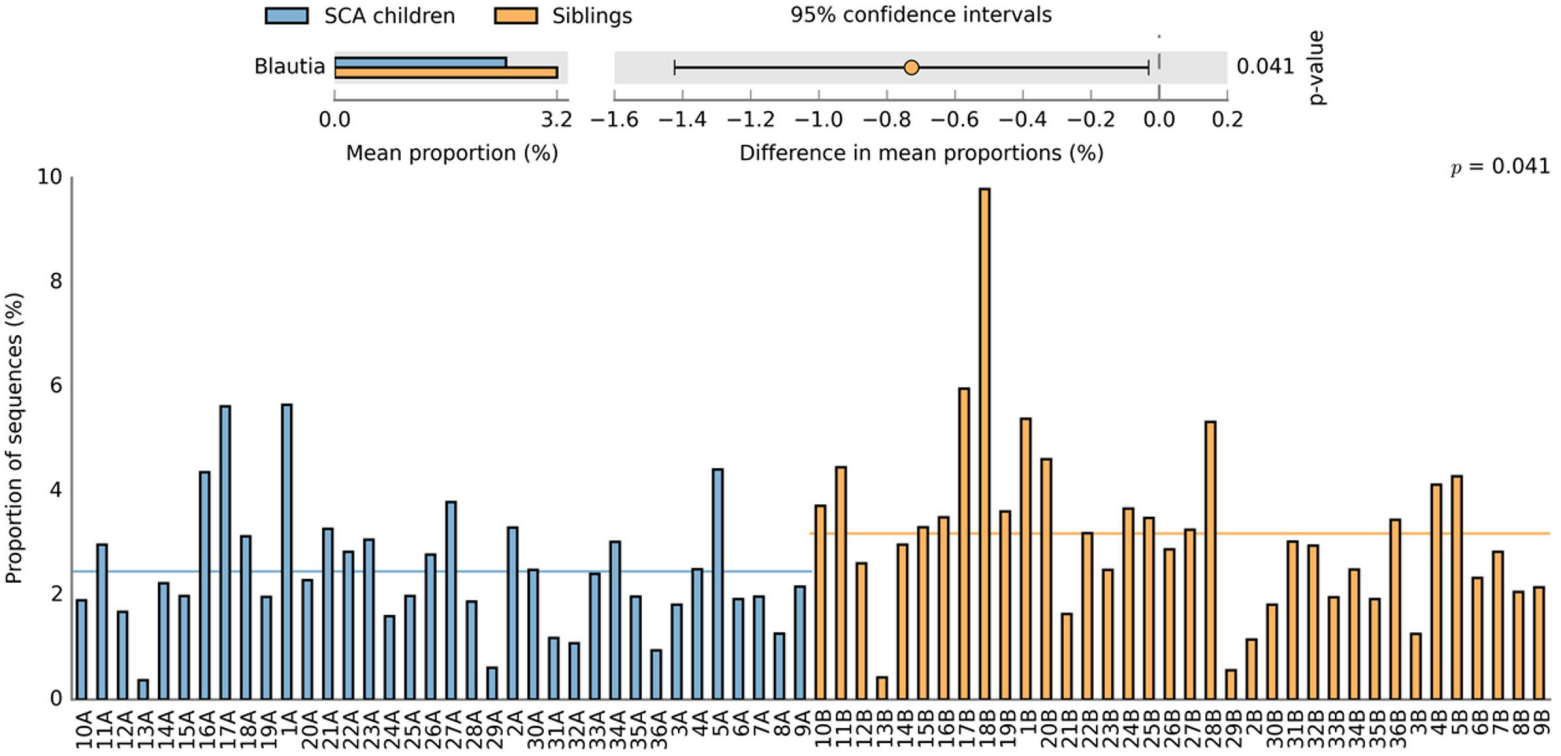
(A) Extended error bar plot and (B) Bar plot with the proportion of sequences of the genus *Blautia* in each sample. The results were filtered using a *p*‐value of 0.05 with a Welch's *t*‐test, effect size of 0.50 threshold and retaining unclassified reads in STAMP software

The microbiota data analysis of the most prevalent bacterial genera in SCA children in relation to their corresponding sibling is represented in Figure 5. When we alter the filtering parameters, the only genus of the top 10 that had significant differences in the proportion of sequences between the two groups was *Blautia* (*p* = 0.041). This particular analysis is represented in more detail in Figure 6.

### 3.4 Disease severity and microbial diversity

**FIGURE 7:**
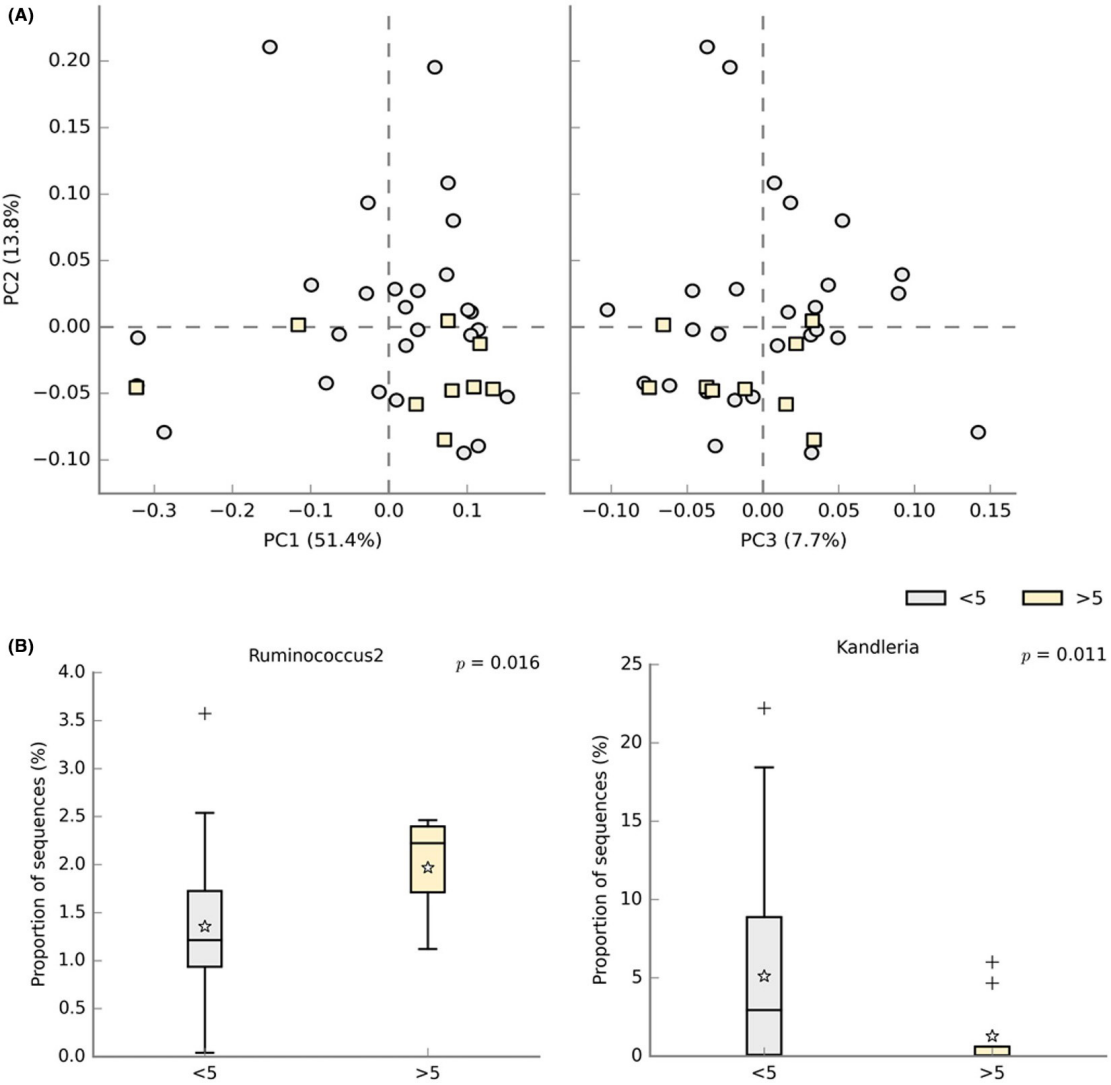
Microbiota analysis plots of the 36 SCA children grouped in relation to their HbF group: (A) Principal component analysis (PCA) plots, where the percentage of variation explained by the principal coordinates is indicated on each axis. Grey circles represent the SCA children with <5% of HbF (*n* = 27) and yellow squares with above 5% (*n* = 9); (B) Bar plots representing the significant genus: *Ruminococcus* and *Kandleria*. The star symbol indicates the mean and the plus symbol the outliers. This analysis was performed with Welch's t‐test and an effect size filter of 0.5

Of the 36 individuals with SCA, 9 of them exhibit higher values of HbF, more than 5%. Clustering analysis in Figure 7A appears to show a separation in the HbF >5% samples. PC1represents 51.4% of the total variability, while PC2 explains a lower fraction of the total variability (13.8%), and PC3 with 7.7%. Additionally, it is possible to notice that the relative abundance of *Ruminococcus* was higher in the group with more than 5% of HbF (*p* = 0.016). In contrast, the bacteria *Kandleria* was less prevalent in this same group, which is supported by a low *p*‐value of 0.011 (Figure 7B).

## 4 DISCUSSION

The alpha diversity in SS genotype was slightly lower than in the control group, and the SS individuals with higher levels of HbF appeared to have a less diverse microbiota. However, no significant differences were found. There were cases of malnutrition among the enrolled participants, as expected for untreated children with SCA living in low and middle‐income countries. However, we did not expect to encounter significant differences in BMI percentile values and BMI group, in relation to the siblings. Although this parameter had no effect on diversity, it may have an influence on microbial composition.

We found that patients of the SCA group had a higher prevalence of the genus *Clostridium XI*. This genus is divided into 19 clusters, and within the cluster XI, it included some harmful bacteria such as *C. difficile, C. sordellii* and *C. bifermentans* ^21^. Typically, patients with a *C. difficile* infection have higher numbers of *Clostridium* XI.^22^ Additionally, it has been proven that the relative increase in *Clostridium* cluster XI is associated with intestinal dysbiosis.^23^

Members of the taxonomic genus *Blautia* have a major impact on human health, due to their role in indigestible carbohydrate degradation and production of short‐chain fatty acids (SCFAs), which act as metabolic mediators between the microbiota and the host.^24^ This bacteria is dominant in a healthy gastrointestinal tract, representing between 2% and 8% of the human gut microbiota, and has a correlation with several host physiological dysfunctions, such as obesity, diabetes, cancer and inflammatory diseases.^24^, ^25^ For example, it has been shown that its abundance is reduced in children with type 1 diabetes and in patients with colorectal cancer.^26^ In our population, we noticed a lower abundance of *Blautia* in the SCA children than in the sibling group. Interestingly, *Blautia* is also known for its contribution in alleviating metabolic and inflammatory diseases and for its potential as a probiotic.^25^

We noticed that five different species of *Prevotella* were more prevalent in the sibling groups. *Prevotella* strains are classically recognized as commensal bacteria due to their abundance in the healthy human body and rare involvement in infections.^27^ However, some strains may exhibit pathobiontic properties and lead to induction of neutrophil dysfunction, which causes chronic inflammation.^27^

*Ruminococcus* abundance was higher in the group of patients with higher levels of HbF, something that is associated with a better prognosis in this disease. Indeed, we noticed that these children had a lower number of transfusions and hospitalizations per year when compared to the children with less than 5% of HbF. The bacteria from the *Ruminococcus* genus are considered to be important gut microbial mutualists, able to degrade and convert complex polysaccharides into a variety of nutrients for their hosts.^28^ *Ruminococcus* species are defined as strictly anaerobic, gram‐positive and require fermentable carbohydrates for growth.^29^ It has been demonstrated that some species, namely *R. albus, R. callidus* and *R. bromii*, have a lower abundance in faecal samples of Crohn's disease patients than in healthy control subjects.^30^

In this work, we also observe a substantial difference in the abundance of the genus *Kandleria*, being higher in the patients with lower HbF levels. These bacteria seem to produce lactate as their fermentation end products.^31^ Unfortunately, little is known about their role in human health.

Our results were not in agreement with other studies, but we need to take into account that are plenty of variables that could be influencing the microbiota. Most of the studies performed in SCD were in mice and the few in humans, are generally in adults from other geographical locations. For example, a recent publication^32^ reported 20 bacterial families significantly different between a group of 14 SCD adults and 14 healthy controls. But, none of these bacterial families reported seem to be significant in our population. Since there is a lack of studies linking the gut microbiome to SCD, further research is needed and could support the reproducibility of current findings.

It is worth mentioning that heavy use of antibiotics at early ages could affect greatly the evolution of the gut microbiome and normally it is a common practice for SCD patients, specially at prophylactic doses. However, in our particular population, given the scarcity of medicines and health resources in Angola, most children have limited access to antibiotics. In fact, one of the inclusion criteria was that there had been no antibiotic exposure in the three months preceding sample collection. As a result, we believe it had no effect on the scale of dysbiosis reported in our findings.

In conclusion, we have presented the first microbiota analysis in an Angolan paediatric population with SCD and provided a detailed view of the microbial differences between patients and healthy controls. However, more research is needed in order to assess the influence of specific bacteria and accurately determine the characteristics of the gut microbiota.

The main importance of this type of studies resides with the fact that some new clinical approaches could be used instead of the current treatment (hydroxyurea), which has several side effects. In order to mitigate the severe symptoms associated with SCD, some interruptive clinical approaches could be used for: reducing the intestinal microbial density by using antibiotics, restoring the intestinal barrier with the help of certain prebiotics or even replenishing the normal intestinal microbial composition with probiotics or faecal microbiota transfer.^16^, ^33^ Studying and optimizing these future therapies could someday have a major impact on the SCD patient's care management.

## AUTHOR CONTRIBUTIONS

**Mariana Delgadinho:** Conceptualization (equal); formal analysis (equal); investigation (equal); methodology (equal); writing – original draft (equal). **Catarina Ginete:** Formal analysis (equal); investigation (equal); methodology (equal); writing – review and editing (equal). **Brigida Santos:** Data curation (equal); formal analysis (equal); investigation (equal); methodology (equal); writing – review and editing (equal). **Joana Mendes:** Data curation (equal); investigation (equal); methodology (equal); writing – review and editing (equal). **Armandina Miranda:** Data curation (equal); investigation (equal); methodology (equal); writing – review and editing (equal). **Jocelyne Neto de Vasconcelos:** Conceptualization (equal); funding acquisition (equal); methodology (equal); writing – review and editing (equal). **Miguel Brito:** Conceptualization (lead); data curation (equal); formal analysis (equal); funding acquisition (equal); investigation (equal); methodology (equal); supervision (lead); writing – review and editing (lead).

## CONFLICT OF INTEREST

The authors declare that there is no conflict of interest regarding the publication of this article.

## PATIENT CONSENT STATEMENT

Written informed consents were obtained from the children's legal guardians prior to inclusion in the study.

## Data Availability

Data is available from the corresponding author on reasonable request.
